# Microdroplet Actuation via Light Line Optoelectrowetting (LL-OEW)

**DOI:** 10.1155/2021/3402411

**Published:** 2021-12-23

**Authors:** Christoph Doering, Johannes Strassner, Henning Fouckhardt

**Affiliations:** Integrated Optoelectronics and Micro Optics Research Group, Physics Department, Technische Universität Kaiserslautern (TUK), P.O. Box 3049, Kaiserslautern D-67653, Germany

## Abstract

Meanwhile, electrowetting-on-dielectric (EWOD) is a well-known phenomenon, even often exploited in active micro-optics to change the curvature of microdroplet lenses or in analytical chemistry with digital microfluidics (DMF, lab on a chip 2.0) to move/actuate microdroplets. Optoelectrowetting (OEW) can bring more flexibility to DMF because in OEW, the operating point of the lab chip is locally controlled by a beam of light, usually impinging onto the chip perpendicularly. As opposed to pure EWOD, for OEW, none of the electrodes has to be structured, which makes the chip design and production technology simpler; the path of any actuated droplet is determined by the movement of the light spot. However, for applications in analytical chemistry, it would be helpful if the space both below as well as that above the lab chip were not obstructed by any optical components and light sources. Here, we report on the possibility to actuate droplets by laser light beams, which traverse the setup parallel to the chip surface and inside the OEW layer sequence. Since microdroplets are grabbed by this surface-parallel, nondiverging, and nonexpanded light beam, we call this principle “light line OEW” (LL-OEW).

## 1. Introduction

Electrowetting-on-dielectric (EWOD) is a phenomenon [1–13], which has been widely used to change microdroplet curvature. E.g., this way, microfluidic lenses with variable focal lengths can be realized [14–18]. For EWOD, the droplets are usually made of ionized water or an aqueous solution and sandwiched between a top electrode and a dielectric layer above a bottom electrode. (Often, for both the substrate as well as for the superstrate, glass wafers with indium tin oxide (ITO) coatings as electrode layers are used, with the ITO films pointing inwards.) Attached to a voltage source, this setup constitutes a loaded capacitor (with the droplet as the “upper capacitor plate” and the top electrode layer just to contact the droplet). The stored electrostatic energy modifies the surface energy of the droplet on the dielectric layer such that the droplet's contact angle and curvature are reduced (the radius of curvature is increased).

If the bottom electrode is structured and initially only part of the droplet is above the electrode structure, the change of the contact angle will be local. This way, a net force arises parallel to the substrate surface, which drags/pulls the droplet onto the electrode [19–22]. Thus, EWOD can be used to move/actuate droplets, also allowing for splitting and merging/fusion of droplets. This is beneficial for digital microfluidics (DMF) in analytical chemistry, i.e., in lab-on-a-chip applications with droplets (lab on a chip 2.0) [23–33].

The necessity of a structured electrode for EWOD-based droplet actuation has the disadvantage that all desired positions and movements of the droplets have to be known in advance because the bottom electrode has to be prepared lithographically accordingly. If another actuation path becomes necessary, another chip will have to be designed and produced.

Optoelectrowetting (OEW) solves this problem [34–44]. Neither of the electrodes has to be structured. However, another layer, i.e., a photoconductive film, has to be incorporated into the layer sequence between the dielectric layer and the bottom electrode. The capacitance of the layer sequence changes locally, wherever light is impinging and entering the photoconductive layer. With illumination, the photoconductive layer is locally relatively well electrically conductive and can be considered part of the bottom electrode, thus leaving the dielectric layer with its small thickness as the only insulator between the “plates” of the capacitor. Where and when no light impinges, the photoconductive layer is not conductive and has to be considered part of a dielectric two-layer sequence.

This way, the operating point of the module changes locally. It is determined by the EWOD voltage and its modulation frequency as well as by the wavelength and intensity of the impinging light. If parameters are set right, the droplet will move wherever the light spot is moved to, which, e.g., can be manipulated and computer-controlled by a set of external mirrors.

Side note 1: In [45], the authors use OEW in combination with a structured bottom electrode. The structure is a metallic regular close-mesh grid. The droplets can only move along the lines of the grid. However, since each stitch is smaller than the droplets' projected areas, they can be moved to any point on the lab chip. The mesh grid stabilizes the droplets' movements. The OEW light is impinging perpendicularly from below the chip.

OEW comes with two disadvantages. First, it is virtually impossible to optimize the setup (layer materials, layer thicknesses, EWOD voltage and frequency, OEW light intensity and wavelength, and electrical droplet conductivity) in order to guarantee droplet actuation for a wide set of parameters; for example, a slightly different OEW light intensity or wavelength might change the whole situation. Secondly, the light usually impinges onto the lab chip perpendicularly, and thus the light source and optical elements will block the space above or below the lab chip. One example for an application, where this is a restriction, is formed by SERS (surface-enhanced Raman scattering) [46], i.e., molecule detection by Raman scattering spectroscopy with signal enlargement due to local field enhancement, especially in DMF [47–49] (the enhancement is achieved by the attachment of the droplets of diluted molecules, e.g., to metallic surfaces or clusters).

Side note 2: In [50], the absorption of the photoconductive layer, in that case, made from titanium oxide phthalocyanine (TiOPc), is enhanced and controlled by plasmonic nanoparticle incorporation into the TiOPc layer. Thus, the metallic particles are not intended to enhance scattering, but performance of the photoconductive layer.

An improvement might come from light, which is entering the chip from the side. A principle possibility might be to couple the OEW light into a film waveguide, which has to be incorporated into the layer sequence of the lab chip. The guided film wave, divergent in the other lateral dimension, might provide for a funnel-like path of any droplet between two regions on the chip with different light intensities.

However, as we came to know in the work leading to [51], it is also virtually impossible to fulfill the requirements of OEW and film wave guiding simultaneously. To give an example, on one hand, the light has to be absorbed to some extent in the photoconductive layer for the latter's very purpose. Yet, on the other hand, not only the film wave divergence but also the absorption will result in different light intensities in different parts of the lab chip. Thus, OEW will not work equally well across the chip.

**Figure 1:**
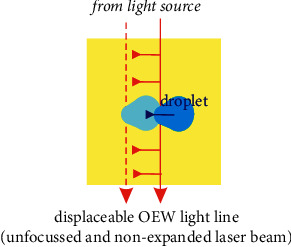
Sketch in top-view of a lab chip in the light line OEW (LL-OEW) case. The light line touches and actuates/moves the droplet. A sideways movement of the light line drags/pulls the droplet to the side as well. Depending on exact parameters, the droplet might also be pushed [44].

In this contribution, we report on a successful *variation* of the last concept, which we call “*light line OEW*” (LL-OEW) because a nondivergent, nonexpanded laser beam is passing the lab chip from side to side. And the droplet is not moved along this light beam, but rather perpendicular to it whenever the light beam/line as a whole is moved into that direction; see Figure 1.

## 2. Further EWOD and OEW Details [1–13, 34–44]

For EWOD, a *d*_diel_ thick dielectric layer forms the insulator of the capacitor. Its loading by applying a voltage *V* leads to the storage of electrostatic energy, which reduces the surface energy/tension *γ*_*l,diel*_ of the liquid droplet on the dielectric layer to an effective value:

(1) $$\begin{array}{c} \gamma_{l,\text{diel}}^{\text{eff}}=\gamma_{l,\text{diel}}-\frac{\varepsilon_{0}\varepsilon_{\text{diel}}}{2d_{\text{diel}}\gamma_{la}}\cdot V^{2}=\gamma_{l,\text{diel}}-\frac{C}{2\gamma_{la}A}V^{2}, \end{array}$$

where *C* is the (local) capacitance, *A* the projected area of the droplet's part above the electrode, *ε*_0_ the dielectric constant, *ε*_*diel*_ the dielectric number of the dielectric layer, and *γ*_*la*_ the surface energy of the interface between liquid/droplet and ambient (often air). The contact angle *θ* of the droplet on the dielectric layer (when compared to Young's angle *θ*_*0*_ for zero voltage) is always reduced according to the Young–Lippmann equation:

(2) $$\begin{array}{c} \cos\theta =\cos\theta_{0}+\frac{\varepsilon_{0}\varepsilon_{\text{diel}}}{2d_{\text{diel}}\gamma_{la}}\cdot V^{2}=\cos\theta_{0}+\frac{C}{2\gamma_{la}A}\cdot V^{2}. \end{array}$$

Since in equation (2), the contact angle is a function of the voltage squared, the polarity of the voltage has no effect, and even an alternating voltage *V* = *V*_*ac*_ is typically employed. For frequencies of ≈100 Hz and more, the droplets cannot follow. Then, the quantity *V* = *V*_*ac*_ has to be regarded as an effective voltage *V*_eff_. We typically use a frequency of ≈1 kHz for EWOD and ≈50 Hz for OEW. According to equations (1) and (2), the application of a voltage always leads to a reduction in surface energy/tension *γ*_*l,*diel_ ⟶ *γ*^*eff*^_*l,*diel_ and thus to a decrease in the contact angle and an increase in the radius of curvature (i.e., weaker curvature) of the droplet. As stated in Section 1, if the latter were used as a tunable plano-convex lens, its focal length could be increased this way.

EWOD can also be employed for droplet actuation if the bottom electrode layer of the capacitor is structured, and the droplet is only partially covering an electrode pad. Then, the ionic droplet will experience a net force moving the droplet further onto the biased pad. However, with several electrically nonjoined small electrode pads adjacent to each other, the droplet might only move on, if its projected area were larger than any pad and always touched two pads. (Of course, the timely loading of the pads has to be controlled appropriately as well.)

Typically, the necessary electrical bias is a rectangular alternating voltage with a positive- and a negative-valued half period.

For EWOD, an alternating voltage is not necessary. But, it also prevents charging of the chip with respect to the surroundings and an electrolytical dissociation of the aqueous solution.

**Figure 2:**
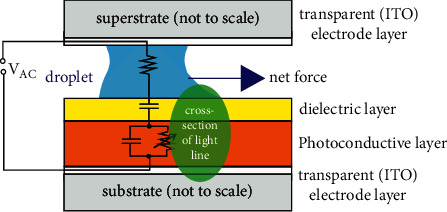
Cross-sectional sketch of OEW setup with equivalent electrical circuit. The OEW light is already illustrated for the LL-OEW case with the light line with (in our case) elliptical cross section entering the chip from one side (here from the front facet). It has to be kept in mind that the equivalent circuit has different values for the capacitances and impedances at sites with or without light incidence. Thus, the equivalent circuit has to be understood as locally valid. The modulation frequency has an influence on the impedances.

On the contrary, for OEW, the alternating voltage is necessary because the interplay of the complex impedances of the dielectric layer, the photoconductive layer, and the path resistance of droplet and contact layers allows for the manipulation of the operating point of the OEW chip. These impedances are dependent on the modulation frequency of the alternating voltage. And variations of the voltage and frequency allow for finding the best operating point, i.e., the parameters, which result in the strongest OEW forces. Therefore, the chip has to be considered as an electrical circuit, sketched with its equivalent elements in Figure 2. The equivalent circuit has locally different values for the capacitances and resistors at sites with and without light incidence.

Moreover, Moench and others even proved that by the right choice of modulation frequency and voltage amplitude, the droplet cannot only be pulled/dragged by the light spot, but even pushed [44].

The modified Young–Lippmann equation to be applied is

(3) $$\begin{array}{c} \cos\;\theta =\frac{\cos\;\theta_{0}\left(1-2\gamma /3\gamma_{la}\right)+\left(C_{\text{dpc}}/2\gamma_{la}A\right)\cdot {\left|V_{\text{dpc}}\right|}^{2}}{1+\left(\gamma /3\gamma_{la}\right)\cos\;\theta_{0}}, \end{array}$$

with *C*_dpc_ as the capacitance of the double-layer (dielectric plus photoconductive layer) and *V*_dpc_ as the voltage drop across the double-layer. Both the denominator as well as the parameter *γ* in the above equation are related to the Laplace pressure and its case-dependent significance, respectively [52, 53], which get strong for microdroplets with volumes well below 1 *μ*l; the Laplace pressure (related to the strong surface curvature of small droplets) works against the reduction of surface tension.

The abovementioned interplay of the complex-valued impedances *Z* is incorporated in the voltage drop *V*_dpc_ according to

(4) $$\begin{array}{c} V_{\text{dpc}}=\frac{Z_{\text{dpc}}}{Z_{\text{dpc}}+R_{\text{path}}}\cdot V, \end{array}$$

with the supply voltage *V*, where *R*_path_ is the overall ohmic path resistance of the droplet itself and the supply lines (electrode layers).

Mathematical notes: Since the impedance *Z*_dpc_ is a complex-valued quantity, in principle, the voltage *V*_dpc_, too, has to be considered a complex-valued quantity according to equation (4). Thus, we have taken the absolute value squared of this voltage and not just the square in equation (3). Moreover, if the definition of an effective voltage *V*_eff_ is applied in its version with a complex-valued voltage and current, one will automatically get the absolute value (squared) of the voltage in equation (3).

Equation (3) has to be applied in the cases with and without illumination to calculate the change of the contact angle through illumination as compared to the case without illumination (which might be called the case of pure EWOD):

(5) $$\begin{array}{c} \Delta \theta =\theta_{\text{illumination}}-\theta_{\text{no}-\text{illumination}}. \end{array}$$

Coarsely speaking, negative contact angle changes Δ*θ* < 0 are related to pull actuation of the droplets and Δ*θ* > 0 to push actuation. Which situation applies strongly depends on the applied voltage and its modulation frequency (and all other parameters).

The OEW force can be estimated to be

(6) $$\begin{array}{c} F=\frac{C_{\text{dpc},\text{illumination}}}{2A}\cdot {\left|V_{\text{dpc,illumination}}\right|}^{2}\cdot L, \end{array}$$

with *L* as the length of the illuminated portion of the droplet's contact line. Light line OEW (LL-OEW) has the advantage over OEW that this portion is longer, such that the overall actuation force is larger than for usual OEW with perpendicular light incidence. Nevertheless, the forces are small; in our LL-OEW case, the maximum force amounts to just 0.75 *μ*N.

As mentioned above, we follow the notion that a negative contact angle change is related to pulling, while a positive change stands for pushing. This is not strictly true, because the pure EWOD case (i.e., the case without light) is already related to a pulling force. This force has to be overcompensated to initialize pushing. But, the applied notion eases explanation.

## 3. Lab Chip Layout, Electrical and Optical Setup

**Figure 3:**
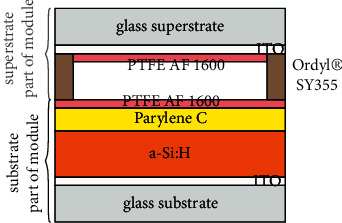
Cross-sectional sketch (not to scale, colors not related to spectral transmission characteristics) of layer sequence of chip/module. From bottom to top: 1.1 *µ*m thick glass slide (substrate), (140 ± 20) nm thick ITO electrode layer, (790 ± 10) nm thick a-Si : H photoconductive layer (dielectric number *ε*_pc_ = 11.8), (360 ± 10) nm thick Parylene C dielectric layer (*ε*_diel_ = 3.1), approx. 10 nm thick PTFE AF 1600 hydrophobic layer, 100 *µ*m to 250 *µ*m thick Ordyl® SY 355 spacer, approx. 10 nm thick PTFE AF 1600 hydrophobic layer, (140 ± 20) nm thick ITO electrode layer, and 1.1 *µ*m thick glass slide (superstrate).

The OEW chip consists of a substrate and a superstrate, each with an area of 25 mm × 25 mm. A cross-sectional view of the chip is given in Figure 3 (with typical parameter values in the caption).

The substrate part is formed of several layers deposited onto an ITO-coated glass slide from Merck KGaA, Darmstadt, Germany. The glass slide has a thickness of 1.1 *µ*m, the ITO layer is (140 ± 20) nm thick, and the optical transmission of the ITO glass slide is about 87% in the visible spectral range. A hydrogenized amorphous silicon (a-Si : H) layer is deposited onto the ITO-coated bottom glass slide by plasma-enhanced chemical vapor deposition (PECVD) with a PlasmaLab 80 Plus machine from Oxford Instruments Plasma Technology, Bristol, UK. This photoconductive a-Si : H layer has a thickness of (790 ± 10) nm. It is covered with a (360 ± 10) nm thick dielectric layer consisting of Parylene C and a hydrophobic top layer of approx. 10 nm thin PTFE AF 1600 [54]. The Parylene C raw material is from Specialty Coating System Inc., Indianapolis, IN, USA and is deposited by chemical vapor deposition using a PDS 2010 also from Specialty Coating System Inc. PTFE AF 1600 is from Sigma Aldrich, Taufkirchen, Germany, and is spin-coated using a Delta BM Gyrset from SÜSS MicroTech, Garching, Germany.

The superstrate part consists of an ITO-coated glass slide of the same type, and a 100 to 250 *µ*m thick (depending on designated droplet size) Ordyl® SY 355 film is used as the spacer. Ordyl® SY 355 is a dry film resist from ElgaEurope s.r.l, Milano, Italy. The thickness of one single fabricated Ordyl® SY 355 layer is 50 *µ*m. The dry film resist is applied to the substrate by hot-roll lamination of two to five layers (giving the total thickness of 100 to 250 *μ*m) and structured photolithographically. Like the substrate part, the superstrate part is hydrophobized with an approx. 10 nm thin, porous PTFE AF 1600 layer. In the superstrate case, this layer is deposited directly onto the ITO layer.

The 10 nm thin PTFE AF 1600 layers are porous, such that the electrical contact of the droplets to the dielectric layer and to the top electrode layer does not deteriorate.

To some extent, the optimization of the layer thicknesses has been a matter of trial and error, in combination with calculations in advance incorporating the dielectric numbers *ε*_diel_ and *ε*_pc_ of the materials for the dielectric and the photoconductive layer (at small frequencies). The aim has been to achieve capacitances and impedances of the layer sequence, which allow for a strong interplay, and to find a situation, where a small change in voltage or frequency has a strong impact, thus maximizing forces on the droplets.

The determination of the layer thicknesses achieved in a chip production run is performed by interpolation of data from calibration runs, in which the material deposition time is varied, and the resulting layer thicknesses are measured, e.g., with a surface profiler comparing a part of the wafer with deposited layer to another part, which has been obstructed during deposition, by measuring the step from one to the other.

**Figure 4:**
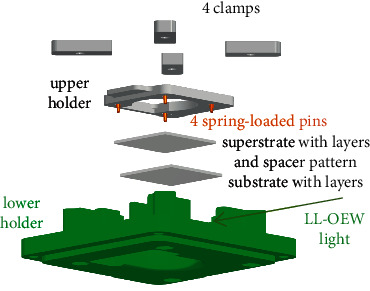
Exploded assembly drawing of setup with lab chip parts. The LL-OEW light is entering the setup from the front right.

The droplets of 2% NaCl aqueous solution with volume in a range between 0.2 and 0.8 *μ*l are placed within an area of 10 mm × 10 mm within the spacer pattern. Then, the substrate and the superstrate part of the module are joined by spring-loaded pins 0906-1-15-20-75-14-11–0 from Mill-Max Manufacturing Corp., Oyster Bay, NY, USA, and clamps.  Figure 4 contains an exploded assembly view of the mechanical bond.

A rectangular alternating voltage in the range of ± (30…70) V at a frequency of 50 Hz is applied for OEW and LL-OEW.

In both cases, the illumination is done with a laser diode L520P50 from ThorLabs GmbH, Bergkirchen, Germany. The laser diode has an emission wavelength of *λ* = 520 nm, and the light intensity is reduced by a neutral density filter to a continuous wave power of *P* = 150 nW on the chip. The laser beam (collimated with a lens right behind the laser diode) is neither focused nor expanded further down along its path. The light beam has an elliptical cross section with semiaxes of 0.75 and 0.27 mm lengths, giving an intensity of 0.74 W/m^2^. Higher intensities do not necessarily mean better OEW actuation.

## 4. Droplet Movement with Usual OEW

**Figure 5:**
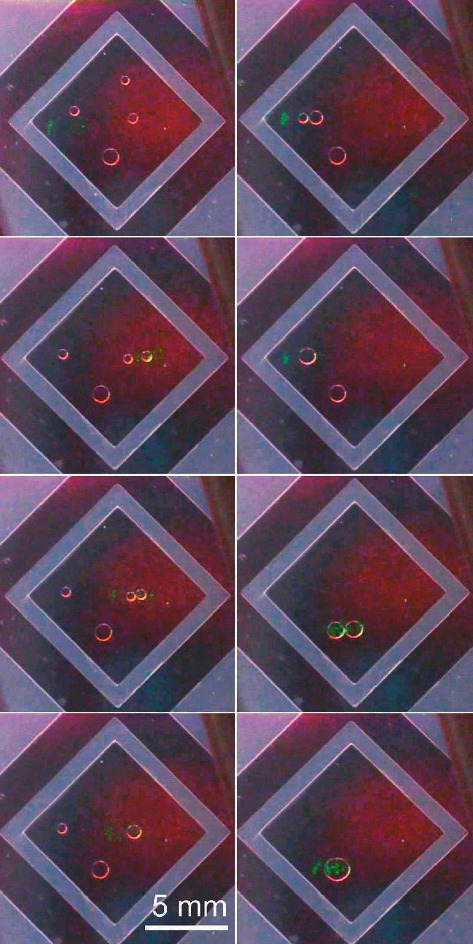
(OEW) Frames from a video (to be read columnwise from top to bottom and from left to right) showing the droplet movement actuated by a perpendicularly incident laser beam (from below here). The droplets are moved towards each other one after the other and merged/fused. The transparent spacer pattern appears light gray here. The darker regions beyond the spacer are areas like those inside the spacer pattern, but not relevant for the purpose of this OEW actuation test. ((V)_ac_ = ±  40 V @ 50 Hz).

To demonstrate the usual OEW for comparison, Figure 5 shows different frames from a video on droplets' movements and merging. The applied voltage is *V*_ac_ = ± 40 V at 50 Hz. A laser spot is directed *perpendicularly* onto the respective droplet from below by the movement of a manually controlled external mirror.

## 5. Droplet Movement with Light Line (LL-) OEW

As stated before, given the OEW chip is used for applications in DMF, the space below and above the lab chip should not be obstructed by the OEW light source, mirrors, or lenses. Therefore, illumination is performed from the side of the lab chip.

**Figure 6:**
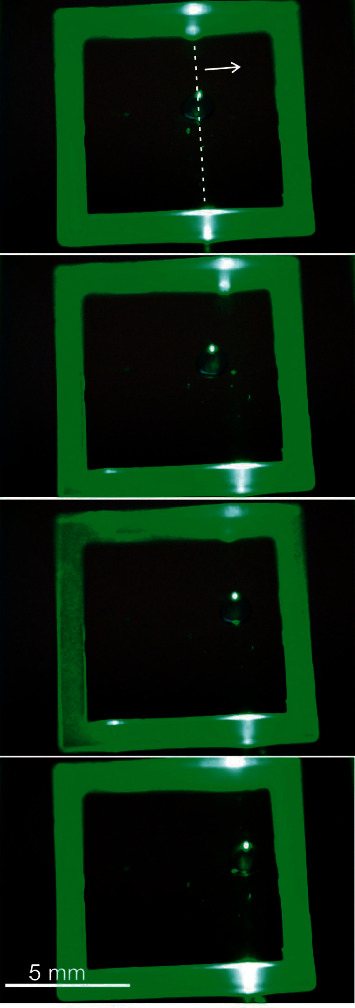
(LL-OEW) Frames from a video showing the droplet movement as a consequence of the movement of the light line. The droplet of 1 mm radius is actuated with a voltage of ±48 V at 50 Hz. The spacer appears green due to the scattered light from the OEW laser diode source. The surface-parallel light line can be identified by higher scattered intensities (even within the dark area) and reflexes. The droplet volume is 0.8 *μ*l here.

Figure 6, again with frames from a video, shows the module in operation. The applied rectangular alternating voltage is *V*_ac_ = ±  48 V @ 50 Hz. Due to its nearly transparent material, the spacer appears green due to the scattered LL-OEW light. The light line enters the lab chip *surface-parallel* from the upper side of the video frames and can be identified by higher scattered intensity and reflexes. The droplet is successfully grabbed and moved by shifting the light line. Actually, in this case, the droplet is pushed rather than pulled by the light line.

Both for Figure 5 as well as for Figure 6, the videos are provided as supplementary materials to this contribution (see below).

In the videos, the movements of the droplets are rather slow due to the slow shift of the light line, which is actuated manually. However, the EWOD response time of the droplets with a similar layer sequence is < 40 ms and possible droplet translation speed is 12 mm/s, as we have shown in [55].

**Figure 7:**
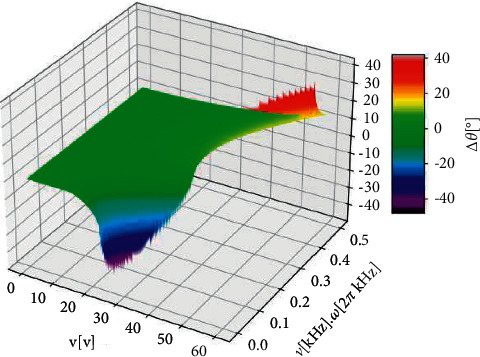
Calculated 3D color-plot of the contact angle change Δ*θ* for variable supply voltage (V) and variable modulation frequency *ν*. The values of the calculated contact angle change Δ*θ* are given in the third dimension, but are also color-coded (see color scale on the right). The other parameters are very similar to those from the situation for Figure 6: (i) droplet's area above the electrode (projected on the electrode): *A* ≈ 3.14 10^−6^m^2^ with an illuminated fraction of 10%; (ii) surface energy of the liquid droplet in ambient/air: *γ*_*la*_ ≈ 0.07 N/m; (iii) Ohmic path resistance beyond double-layer: *R*_Path_ = 5.0 MΩ; (iv) Ohmic resistance of photoconductive a-Si :H layer without illumination: 12 MΩ; (v) Ohmic resistance of photoconductive a-Si :H layer with illumination: 0.12 MΩ.

Videos and frames from them for other voltages and frequencies would look very similar. Thus, to shed some more light on the complex situation, we give the results of calculations regarding the setup from Figure 6. The additional parameters are given in the figure caption of Figure 7; the figure itself shows the calculated results. The contact angle change is plotted as a function of supply voltage *V* and modulation frequency *ν*.

For small frequencies and larger voltages, there is an area of undefined contact angle changes, in which no values are plotted. This is the area, where the right side of equation (3) is larger than 1 and, thus, out of bounds for any cosine function. In physical reality, this range is related to a saturation of the contact angle. For voltages below about ±39 V, there is droplet pulling (Δ*θ* < 0) for very small frequencies below ≈200 Hz and nearly no pulling and no pushing otherwise. For higher voltages, i.e., above about ±39 V, there is no pulling, but pushing (Δ*θ* > 0) for frequencies still well below 1 kHz (on the contrary to the findings in [44]). The maximum values of the contact angle change for pulling and pushing are similar for this set of parameters with amounts of ≈40°.

The calculated results reveal that the ranges of voltage and frequency, where the contact angle changes are not negligible, are very narrow. A change of the voltage by ±2.5 V or a change of the frequency by ±25 Hz might result in just minor contact angle changes (or mathematically even undefined values). This is another disadvantage of OEW (and LL-OEW) as compared to EWOD. The freedom of droplet actuation paths is paid for by the narrow window of optimized parameters.

Another problem is that not only the supply voltage and its modulation frequency have an impact, but also all other parameters, even the droplet's electrical conductance (leading to a specific ohmic resistance), but also the droplet's size. The smaller a droplet, the stronger is the Laplace pressure. Moreover, the droplet size also determines the projected area *A* of the droplet and the length *L* of the illuminated portion of the contact line. Thus, any optimization of the chip can only be done considering a certain range of droplet sizes (volumes and projected areas). For another range, another chip has to be prepared after appropriate calculations. – In practice, the smallest allowable droplet diameter is given by the distance between the substrate with its layers and the superstrate with its layers (in our case, 100 to 250 *μ*m), because the droplet has to touch both the dielectric layer (with PTFE on top) and the upper electrode layer (with PTFE below).

Despite their problems, OEW and LL-OEW add some flexibility to DMF module use.

## 6. Conclusions

We have presented a new concept, called light line optoelectrowetting (LL-OEW), to use OEW for droplet actuation, possibly in digital microfluidics (DMF) for any application in analytical chemistry on labs on a chip (lab on a chip 2.0). The OEW light is traversing the lab chip sideways, thus keeping the space above and below the chip free of optical components and the (laser) light source. This is a necessity for applications, where the droplets also have to be monitored/manipulated/analyzed otherwise.

Light line optoelectrowetting (LL-OEW) adds another opportunity to the EWOD/OEW toolbox.

## Data Availability

Videos to Figures 5 and 6 are given in the link in section “Supplementary Materials.” Further data that support the findings of this study are available from the corresponding author upon reasonable request.

## Supplementary Materials

Supplementary Materials Videos to Figure 5 (usual OEW) and Figure 6 (LL-OEW) can be found “https://seafile.rlp.net/d/2626c044d4b04d43bfe7/”.

## References

[1] Berge B. (1993). Électrocapillarité et mouillage de films isolants par l’eau. *Comptes Rendus de l’Academie des Sciences Serie II B Mecanique-Physique-Chimie-Astronomie*.

[2] Vallet M., Vallade M., Berge B. (1999). Limiting phenomena for the spreading of water on polymer films by electrowetting. *The European Physical Journal B*.

[3] Verheijen H. J. J., Prins M. W. J. (1999). Reversible electrowetting and trapping of charge: model and experiments. *Langmuir*.

[4] Buehrle J., Herminghaus S., Mugele F. (2003). Interface profiles near three-phase contact lines in electric fields. *Physical Review Letters*.

[5] Shapiro B., Moon H., Garrell R. L., Kim C.-J. C. (2003). Equilibrium behavior of sessile drops under surface tension, applied external fields, and material variations. *Journal of Applied Physics*.

[6] Kang K. H. (2002). How electrostatic fields change contact angle in electrowetting. *Langmuir*.

[7] Jones T. B. (2002). On the relationship of dielectrophoresis and electrowetting. *Langmuir*.

[8] Moon H., Cho S. K., Garrell R. L., Kim C.-J. C. (2002). Low voltage electrowetting-on-dielectric. *Journal of Applied Physics*.

[9] Mugele F., Baret J.-C. (2005). Electrowetting: from basics to applications. *Journal of Physics: Condensed Matter*.

[10] Jones T. B. (2005). An electromechanical interpretation of electrowetting. *Journal of Micromechanics and Microengineering*.

[11] Mugele F., Buehrle J. (2007). Equilibrium drop surface profiles in electric fields. *Journal of Physics: Condensed Matter*.

[12] Berthier J., Dubois P., Clementz P., Claustre P., Peponnet C., Fouillet Y. (2007). Actuation potentials and capillary forces in electrowetting based microsystems. *Sensors and Actuators A: Physical*.

[13] Wang K.-L., Jones T. B. (2005). Saturation effects in dynamic electrowetting. *Applied Physics Letters*.

[14] Berge B., Peseux J. (2000). Variable focal lens controlled by an external voltage: an application of electrowetting. *The European Physical Journal E*.

[15] Gabay C., Berge B., Dovillaire G., Bucourt S. (2002). Dynamic study of a varioptic variable focal lens. *Proceedings of SPIE*.

[16] Kuiper S., Hendriks B. H. W. (2004). Variable-focus liquid lens for miniature cameras. *Applied Physics Letters*.

[17] Krogmann F., Monch W., Zappe H. (2008). Electrowetting for tunable microoptics. *Journal of Microelectromechanical Systems*.

[18] Zappe H., Duppé C. (2015). *Tunable Micro-optics*.

[19] Pollack M. G., Fair R. B., Shenderov A. D. (2000). Electrowetting-based actuation of liquid droplets for microfluidic applications. *Applied Physics Letters*.

[20] Pollack M. G., Shenderov A. D., Fair R. B. (2002). Electrowetting-based actuation of droplets for integrated microfluidicsElectronic supplementary information (ESI) available: six videos showing droplet flow, droplet dispensing and electrowetting. *Lab on a Chip*.

[21] Kwon Cho S., Moon H., Kim C. (2003). Creating, transporting, cutting, and merging liquid droplets by electrowetting-based actuation for digital microfluidic circuits. *Journal of Microelectromechanical Systems*.

[22] Pei S. N., Wu M. C. On-chip blade for accurate splitting of droplets in light-actuated digital microfluidics.

[23] Cao J., Schneider S., Schultheiß R., Schober A., Köhler J. M., Groß G. A. (2015). From microtiter plates to droplets” tools for micro-fluidic droplet processing. *Microsystem Technologies*.

[24] Li P. C. H. (2005). *Microfluidic Lab-On-A-Chip for Chemical and Biological Analysis and Discovery*.

[25] Fouillet Y., Jary D., Chabrol C., Claustre P., Peponnet C. (2007). Digital microfluidic design and optimization of classic and new fluidic functions for lab on a chip systems. *Microfluidics and Nanofluidics*.

[26] Fair R. B. (2007). Digital microfluidics: is a true lab-on-a-chip possible?. *Microfluidics and Nanofluidics*.

[27] Day P., Manz A., Zhang Y. (2012). *Microdroplet Technology: Principles and Emerging Applications in Biology and Chemistry*.

[28] Banerjee A., Kreit E., Liu Y., Heikenfeld J., Papautsky I. (2012). Reconfigurable virtual electrowetting channels. *Lab on a Chip*.

[29] Banerjee A., Noh J., Liu Y., Rack P., Papautsky I. (2015). Programmable electrowetting with channels and droplets. *Micromachines*.

[30] Choi K., Ng A. H. C., Fobel R., Wheeler A. R. (2012). Digital microfluidics. *Annual Review of Analytical Chemistry*.

[31] Jebrail M. J., Bartsch M. S., Patel K. D. (2012). Digital microfluidics: a versatile tool for applications in chemistry, biology and medicine. *Lab on a Chip*.

[32] Samiei E., Tabrizian M., Hoorfar M. (2016). A review of digital microfluidics as portable platforms for lab-on a-chip applications. *Lab on a Chip*.

[33] Freire S. L. S. (2016). Perspectives on digital microfluidics. *Sensors and Actuators A: Physical*.

[34] Chiou P. Y., Moon H., Toshiyoshi H., Kim C.-J., Wu M. C. (2003). Light actuation of liquid by optoelectrowetting. *Sensors and Actuators A: Physical*.

[35] Chiou P.-Y., Chang Z., Wu M. C. (2008). Droplet manipulation with light on optoelectrowetting device. *Journal of Microelectromechanical Systems*.

[36] Chiou P. Y., Park S.-Y., Wu M. C. (2008). Continuous optoelectrowetting for picoliter droplet manipulation. *Applied Physics Letters*.

[37] Park S.-Y., Chiou P.-Y. (2011). Light-driven droplet manipulation technologies for lab-on-a-chip applications. *Advances in OptoElectronics*.

[38] Valley J. K., NingPei S., Jamshidi A., Hsu H.-Y., Wu M. C. (2011). A unified platform for optoelectrowetting and optoelectronic tweezers. *Lab on a Chip*.

[39] Chiou P.-Y., Park S.-Y. (2012). Single-sided continuous optoelectrowetting (SCEOW) device for droplet manipulation with light patterns.

[40] Yu T.-M., Yang S.-M., Fu C.-Y. (2013). Integration of organic opto-electrowetting and poly(ethylene) glycol diacrylate (PEGDA) microfluidics for droplets manipulation. *Sensors and Actuators B: Chemical*.

[41] Shekar V., Campbell M., Akella S. Towards automated optoelectrowetting on dielectric devices for multi-axis droplet manipulation.

[42] Collier C. M., Hill K. A., DeWachter M. A., Huizing A. M., Holzman J. F. (2014). Optoelectrowetting for continuous microdroplet actuation. *Biophotonics: Photonic Solutions for Better Health Care IV*.

[43] Collier C. M., Hill K. A., DeWachter M. A., Huizing A. M., Holzman J. F. (2015). Nanophotonic implementation of optoelectrowetting for microdroplet actuation. *Journal of Biomedical Optics*.

[44] Krogmann F., Qu H., Mönch W., Zappe H. (2008). Push/pull actuation using opto-electrowetting. *Sensors and Actuators A: Physical*.

[45] Loo J., Pei S. N., Wu M. C. (2021). Co-planar light-actuated optoelectrowetting microfluidic device for droplet manipulation. *Journal of Optical Microsystems*.

[46] Pilot R., Signorini R., Durante C. (2019). Review on Surface-Enhanced Raman Scattering. *Biosensors*.

[47] Benz C., Retzbach H., Nagl S., Belder D. (2013). Protein-protein interaction analysis in single microfluidic droplets using FRET and fluorescence lifetime detection. *Lab on a Chip*.

[48] Meier T.-A., Poehler E., Kemper F. (2015). Fast electrically assisted regeneration of on-chip SERS substrates. *Lab on a Chip*.

[49] Meier T.-A., Beulig R. J., Klinge E., Fuss M., Ohla S., Belder D. (2015). On-chip monitoring of chemical syntheses in microdroplets via surface-enhanced Raman spectroscopy. *Chemical Communications*.

[50] Thio S. K., Bae S., Park S.-Y. (2020). Plasmonic nanoparticle-enhanced optoelectrowetting (OEW) for effective light-driven droplet manipulation. *Sensors and Actuators B: Chemical*.

[51] Heisel C. (2016). Micro-fluidic droplet array actuated via electrowetting.

[52] Jasper W. J., Rasipuram S. (2017). Relationship between contact angle and contact line radius for micro to atto [10^−6^ to 10^−18^] liter size oil droplets. *Journal of Molecular Liquids*.

[53] Jasper W. J., Anand N. (2019). A generalized variational approach for predicting contact angles of sessile nano-droplets on both flat and curved surfaces. *Journal of Molecular Liquids*.

[54] Zhang H., Weber S. G., Horváth I. (2011). Teflon AF materials. *Fluorous Chemistry*.

[55] Strassner J., Heisel C., Palm D., Fouckhardt H. (2018). Microfluidic droplet array as optical irises actuated via electrowetting. *Advances in OptoElectronics*.

