# Supplementary material for: Microdroplet Actuation via Light Line Optoelectrowetting (LL-OEW)
Source: Int J Anal Chem. 2021 Dec 23;2021:3402411. doi: 10.1155/2021/3402411 (PMC8718280; doi:10.1155/2021/3402411)
Supplement: Supplementary Materials — Videos to Figure 5 (usual OEW) and Figure 6 (LL-OEW) can be found “https://seafile.rlp.net/d/2626c044d4b04d43bfe7/”. [file 3402411.f1.docx]

**Supplementary Materials**

Videos to Fig. 5 (usual OEW) and Fig. 6 (LL-OEW) can be found under the following internet link:

<https://seafile.rlp.net/d/2626c044d4b04d43bfe7/>
